# The Comparative Effect of *Citrullus colocynthis* Hydro-Alcoholic Extract and Metformin on Morphometric Ovarian Follicles Disorders in Estradilol Valerate Iinduced-Polycystic Ovary Syndrome Rats

**DOI:** 10.22086/gmj.v0i0.1045

**Published:** 2018-08-11

**Authors:** Mohammad Hossein Barzegar Bafrouei, Homayoun Khazali, Seyyed Mehdi Kalantar, Arezoo Khoradmehr

**Affiliations:** ^1^Department of Physiology, Faculty of Biological Sciences, Shahid Beheshti University, Tehran, Iran; ^2^Research and Clinical Center for Infertility, Yazd Reproduction Sciences Institute, Shahid Sadoughi University of Medical Sciences, Yazd, Iran

**Keywords:** Polycystic Ovary Syndrome, *Citrullus Colocynthis*, Metformin, Follicle

## Abstract

**Background:**

Polycystic ovary syndrome (PCOS) is an endocrine disorder that affects 6-10% of women in reproductive age. The medicinal values of Citrullus colocynthis (CCT) extract stems from its anti-oxidative and anti-inflammatory effects. This study evaluated the comparative effect of CCT and metformin on morphometric ovarian disorders in PCOS rats.

**Materials and Methods:**

Fifty female Wistar rats having 2-3 consecutive estrous cycles during two weeks were divided into five groups (n=10 each group). The rats in the control group only received sesame oil as estradiol valerate solvent, whereas the rats in the sham group were injected intramuscularly with 4 mg/rat estradiol valerate-induced PCOS. Following PCOS induction, the rats in the metformin group received 50 mg/kg metformin orally for three weeks. Rats in the Extract group were treated with 50 mg/kg plant extract for 21 days following the induction with PCOS. Additionally, the rats in metformin+ extract group were treated with the combination of 50 mg metformin plus plant extract for three weeks. The ovaries were removed and were fixed for tissue processing. The slices were stained by hematoxylin-eosin after clearing and hydration. Follicular count and morphometric changes were evaluated in primordial, primary, pre-antral and antral follicles.

**Results:**

The mean diameter of primordial follicle was similar in all groups, but mean diameter of primary follicle decreased in the sham group (145.50± 49.26 µm) compared to metformin (278.76± 156.64 µm), extract (311.70± 147.74 µm) and metformin+ extract groups (265.35± 88.16 µm). The diameter of pre-antral and antral follicles in the sham group were significantly larger than those in the control group, but were not significantly different in all other groups including metformin, extract, and metformin+ extract in comparison with control group (P< 0.05).

**Conclusion:**

In this study, the data has demonstrated that CCT like metformin could improve follicular morphometric disorders in PCOS rats.

## Introduction

Polycystic ovary syndrome (PCOS) is characterized by the presence of hyperandrogenism, oligo or anovulation, and polycystic ovary[1]. PCOS is considered a multifactorial disorder and its mechanism of pathogenesis are not exactly known. Different mechanisms including hyperandrogenism, chronic low-grade inflammatory and insulin resistance have been proposed as the key contributors to the pathogenesis of these disorders [2, 3]. It has been shown that circulating and molecular markers of pro-inflammatory and oxidative stress are strongly correlated with circulating androgens [4]. These findings support the idea that hyperandrogenism induces inflammation which causes hyperglycemia. On the other hand, glucose induces inflammation which promotes overproduction of androgen in the ovary [5, 6]. Overproduction of ovarian androgen is associated with exaggerated biosynthesis of the superfamily of cytochrome P450, number17 (CYP17). Cytochrome P450, number 19 (CYP19) is a complex enzyme consisting of cytochrome P450 aromatase and nicotine amide adenine dinucleotide phosphate cytochrome reductase that converts androgens to estrogens [7]. This enzyme first catalyzes the conversion of pregnenolone and progesterone into 17-hydroxypregnenolone and 17-hydroxyprogesterone, respectively and in the next stages, these steroids are converted into dehydro-epiandrosterone and androstenedione, respectively [8]. Metformin is used as an add-on to insulin in the treatment of type 2 diabetes. To increase fertility and reduce polycystic disturbances, the use of metformin in polycystic individuals has been dramatically increased in the past few years mainly because of the importance of polycystic pathology of insulin resistance [9]. Metformin has been proposed to inhibit complex I (NADH) of the mitochondrial membrane leading to overall reduction in the production of ATP and consequently an increase in AMP/ATP ratio. It is generally accepted that the elevated level of AMP inhibits the production of cAMP which in turn hinders the activity of protein kinase A (PKA), and ultimately suppresses the expression of gene encoding, the enzymes involved in gluconeogenesis. A decrease in the blood glucose level results in low levels of both androgen and insulin plasma levels, consequently improving the condition of hyperinsulinemia [10].

*Citrullus colocynthis* (CCT) Schrad belongs to the Cucurbitaceae family. The plant is widely available in different parts of the world, particularly in Africa and southern part of Asia [11]. The plant is traditionally used for the treatment of some diseases including constipation, leprosy, asthma, bronchitis, jaundice, joint pain, cancer and mastitis [12, 13]. The results from the study of the anti-diabetic effect of the hydro-alcoholic fruit of CCT in normoglycaemic rabbits showed a decrease in blood glucose from 132 to 93 mg/100 mL after 24 h [14]. Some research groups have pointed to the antioxidant activity of CCT extract. By evaluating the antioxidant activity of three isolated flavonoids (isosaponarin, isovitexin, and isoorientin3-O-methyl ether) from CCT extract in the 2, 2-diphenyl-1- picrylhydrazyl (DPPH), a dose-depended behavior was concluded for the anti-oxidative activity of this plant [15-17]. The anti-inflammatory activity of CCT fruit extract (4 mg/kg) has also been studied in carrageenan-induced paw edema model rats, and the results showed that this plant could be used as an anti-inflammation agent in medicine [16, 18, 19].

By using thin layer chromatography technique, Mineea *et al*., isolated and purified quercetin from all parts (root, fruit, leaves, seeds, and stem) of CCT [20]. This quercetin which is a polyphenol from the flavonoid group possesses both anti-inflammatory and anti-proliferation activities. Flavonoids belong to a group of natural polyphenolic compounds which occur in many vascular plants. These compounds are responsible for the color of roots, fruits, leaves, and flowers [21]. All of the flavonoids are synthesized in common biosynthetic pathway which incorporates precursors from both shikimate and acetate-malonate pathways. These pathways are merged at a common step in which a central core of flavonoids as hydroxychalcone is generated [22]. The anti-inflammatory activity of flavonoids is attributed not only to their anti-oxidative effect but also to their inhibitory effect on several key enzymes that metabolize arachidonic acid. Inhibiting the activity of these enzymes reduces the production of prostaglandins and leukotrienes in different cells and thromboxane in platelets [23]. During the inflammatory process, oxidative stress condition is induced by activated phagocytes.

This study aimed to compare the effect of CCT and metformin on morphometric ovarian follicles disorders in PCOS subjects.

## Materials and Methods

### Plant Materials and Extraction

Large amounts of fresh CCT fruits were collected from the city of Ardakan, province of Yazd –Iran. The plant was identified by Dr. Hakimi, from the Department of Biology, Yazd University, and a voucher specimen (C.C-01-01) was deposited in the herbarium of the Faculty of Biology of Yazd University-Iran. Samples were completely washed with deionized water, cleaned with tissue paper and dried under shade to stabilize their compositions. Before preparation of the extract, the dried pulps of fruits were thoroughly grinded with a grinder (Mullenax), and then 250-gram of pulp powder was dissolved in 100 ml water/ethanol mixture (20/80 ml) for one day. The extracts were concentrated using a rotary evaporator at 45°C [24]. The raw extracts (10 gram) were dissolved in freshly prepared normal saline (0.009) to a final holding solution (10 mg/ml) which was later used to provide 150 μl (50 mg/kg) of the extract to the treatment group.

### Animals

This experimental study was carried out on fifty female Wistar rats (aged two months) weighing 180 to 200 gram which were purchased from Pasteur Institute Tehran-Iran. To adopt with laboratory conditions, the rats were transported to the laboratory two weeks prior to the start of the experiments. The animals were kept in polyethylene cages under standard conditions, with ambient temperature 22±1 °C and 12-hour of the dark-light cycle, with free access to daily food and water. The animals which had 2-3 regular estrous cycles for a period of two weeks (monitoring their vaginal smears) were selected for the experiments. PCOS induction was performed by estradiol valerate intramuscular injection where a single dose of 2 mg estradiol valerate (dissolved in 0.2 ml sesame oil) was used for each selected rat. Each injection was followed by monitoring vaginal smear to stabilize persistent vaginal cornification stage. This situation is one of the symptoms of ovarian cystic. PCOS induction process was continued for sixty days.

### Experimental Design

The animals were divided into five groups of 10 animals each. The animals in the control group were only received sesame oil as estradiol valerate solvent. Those in the sham group were injected with estradiol valerate after induction by PCOS. The animals in the metformin group received an average of 50 mg/kg metformin (Aburaihan co. Tehran Iran) for three weeks after induction by PCOS. Those in the plant extract group orally received 50 mg/kg fruit hydro-alcoholic extract of CCT. Metformin+extract group (metformin and extract) was used to evaluate the synergetic effect of extract and metformin in PCOS model. The animals in this group orally received 50 mg/kg of metformin and plant extract. Polycystic ovarian was induced by intramuscularly injecting 2 mg of estradiol valerate dissolved in 0.2 ml sesame oil in sham, metformin, extract and metformin+extract groups.

### Histomorphometric Study

**Figure-1:**
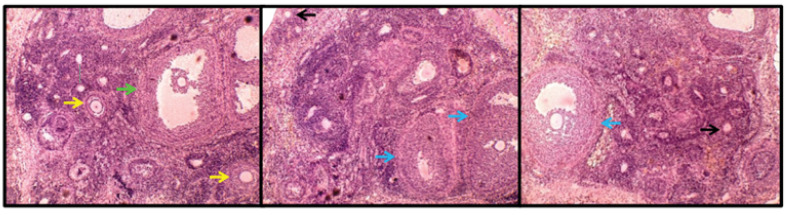
Hematoxylin-eosin staining in sham group: primordial, primary, pre-antral and antral follicles marked by black, yellow, blue and green arrows, respectively (Magnification 200x)

Ovaries of all anesthetized animals were removed, and one of the ovaries was fixed in Bouin fixative for 24 hr for tissue processing. This was followed by dehydration, embedding with paraffin wax and cutting specimens of thickness 6µm on a rotary microtome. After clearing and hydration, the slices were stained by hematoxylin and eosin and were examined with a light microscope (Olympus CX21FS1, Japan). Identification and evaluation of follicular morphometric changes were based on the studies of Istaely at all.,[25] in which the follicles were classified into four groups namely primordial follicles, primary follicles, pre-antral follicles and antral follicles. Primordial follicles consist of oocyte surrounded by one layer flattened pre-granulosa cells. A primary ovarian follicle is a immature follicle comprised of an oocyte surrounded by a single layer of tall, supporting granulosa cells. Pre-antral follicles consist of an oocyte with two or more layers of granulosa cells and no antrum. Finally, antral follicles have follicles with an antral cavity (Figure-1). To count each follicle only once, counting was done where nucleolus was seen within the nucleus of the oocyte. For each follicle, two perpendicular diameters were measured using the micro visible-microscopy software. The measurement was carried out from one basement membrane to the next, and an average of two diameters was calculated for each follicle [26].

### Ethical Approval

All procedures performed in this study that involved experimental animal methods were in accordance with the Ethics Committee of the Research and Clinical Center for Infertility, Shahid Sadoughi University of Medical Sciences, Yazd, Iran, together with 1964 Helsinki declaration and its later amendments or comparable ethical standards (IR-SSU-RSI-REC-1394-1).

### Statistical Analysis

Data analysis was performed using statistical package for the social sciences, version 24.0 (SPSS Inc, Chicago, Illinois, USA). The data were expressed as median (min-max). Kruskal-Wallis test was used for comparison of the data between different groups. The analysis was considered as statistically significant at P-value<0.05.

## Results

**Figure-2:**
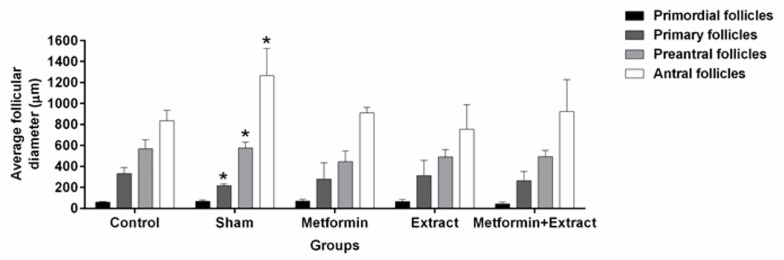
Mean diameter of primordial, primary, pre-antral and antral follicles in all groups. Animals were divided into control (only injected by sesame oil as estradiol valerate solvent), sham (injected intramuscularly 4 mg/rat estradiol valerate- induced PCOS), metformin (orally received 50mg/kg metformin for three weeks after induced PCOS), extract (treated with 50mg/kg plant extract for 21 days after induced PCOS) and metformin and extract (group treated with combination 50mg metformin plus plant extract for three weeks). * shows significant difference of sham group with other groups at a level of P<0.05.

The results of this study demonstrated that the mean diameter of primordial follicles in the sham group (59.47± 5.21 µm) was similar to the other groups including control (67.47±13.40 µm), metformin (64.12±17.86 µm), extract (71.56±20.10 µm), and metformin+extract (63.43± 18.40 µm) (Figure-1). However, the mean diameter of primary follicles in the sham group (145.50± 49.26 µm) was significantly lower than the other groups (Figure-2). Additionally, the mean diameter of pre-antral follicles was significantly increased in the sham group (576.22± 56.47 µm) compared to the other groups (control: 415.29±85.34 µm; metformin: 445.60±102.71 µm; extract: 446.11±70.80 µm; metformin+extract: 426.71±61.65 µm). Furthermore, the results showed that the mean diameter of antral follicles was similar among control (835.74± 99.91), metformin (909.55± 53.89), extract (872.09± 233.33) and metformin+extract (923.85±304.73) groups but was significantly higher in the sham group (1265.02± 260.82 µm). The current results further demonstrated that the mean diameter of oocytes was similar in all groups and in all follicles categories. The mean diameter of oocytes in primordial follicles in control, sham, metformin, extract and metformin+extract groups were 28.80±6.16, 28.86±8.63, 25.73±8.54, 23.55±5.58, 24.64±8.93 µm, respectively. The mean diameter of oocytes in primary follicles in control, sham, metformin, extract and metformin+extract groups were 78.59±17.35, 82.60±25.28, 110.31±46.01, 103.69±28.18, 88.80±44.88 µm respectively. Lastly, the mean diameter of oocytes in pre-antral follicles in control, sham, metformin, extract and metformin+extract groups were 146.88±19.30, 136.76±59.61, 146.39±35.59, 173.55±11.97, and 181.96±88.85 µm, respectively. Also, antral follicles in control, sham, metformin, extract and metformin+extract groups were 327.62±178.59, 193.70±55.84, 177.39±49.17, 188.99±22.07, and 218.62±102.31 µm, respectively.

## Discussion

The results of the present morphometric study showed that the diameter of pre-antral and antral follicles in sham group were significantly larger than those in control, metformin, extract and metformin+extract groups, but were not significantly different among control, metformin, extract and metformin+extract groups. Moreover, these results showed that the mean diameter of primary follicles in sham group was significantly smaller than that of the other groups.

The present results are consistent with results of previous studies. It has been demonstrated that the theca interna of many follicles in women with PCOS is thicker than the normal theca layer [27]. In polycystic ovaries, the theca interna is hypertrophied and contains more layers of steroidogenic cells with excessive androgenic potent. In contrast, the thickness of granulosa cell (estrogen-producing cells) layer is smaller than normal follicles. Moreover, the overproduction of LH and overexpression of LH receptor mRNA in theca interna cells was demonstrated [27]. A possible mechanism for reducing the diameter of primary follicles in sham group compared to other groups is the absence of theca interna cells in these follicles. This can be explained by the fact that these cells are affected by high concentration of LH but not granolusa cells in PCOS subjects [28]. The high ratio of LH/FSH in PCOS can be attributed to the increase in diameter of prenatral and antral follicles in sham group by the thickened theca interna layer and not the granolusa layer. Granolusa cells have numerous receptors for FSH but not LH [29]. The effect of CCT in improving PCOS can also be explained by its anti-inflammatory and anti-oxidant activities since PCOS is a chronic low grade inflammatory and oxidative stress state.

Phytochemical screening of hydromethanolic, crude aqueous and ethyl acetate of CCT extract showed that they contain flavonoids including glucosides, isosaponarin, isovitexin, quercetin and myricetin [30, 31]. According to the studies by Krushangi *et al*. (2015), it can be said that the role of extracts in reducing the diameter of follicles in group IV can be attributed to the inhibitory effect of quercetin flavonoids on phosphatidyl anositol kinase (PI3K). By inhibiting this kinase, the synthesis of protein kinase C is also inhibited, and therefore the synthesis of inflammatory cytokines such as IL-6 and TNF-a is suppressed. By inhibiting the synthesis of these inflammatory cytokines, the growth of these cells, as well as the production of testosterone and LH, are also hindered [32]. Thus, by inhibiting the activity of the cells, the thickness of this layer is decreased, and the overall thickness of the follicle is also reduced compared to the sham group.

## Conclusion

This study has shown that oral administration of CCT hydro-alcoholic extract like metformin improves follicle morphology disorders. These effects can be explained by the potency of flavonoids in attenuation of the pro-inflammatory process.

## Conflict of Interest

All the authors declare that there is no conflict of interest.
